# A Novel ECG Signal Denoising Algorithm Based on Sparrow Search Algorithm for Optimal Variational Modal Decomposition

**DOI:** 10.3390/e25050775

**Published:** 2023-05-10

**Authors:** Jiandong Mao, Zhiyuan Li, Shun Li, Juan Li

**Affiliations:** 1 School of Electrical and Information Engineering, North Minzu University, North Wenchang Road, Yinchuan 750021, China; 2 Key Laboratory of Atmospheric Environment Remote Sensing of Ningxia Province, North Wenchang Road, Yinchuan 750021, China

**Keywords:** ECG signal, sparrow search algorithm, variational modal decomposition, singular value decomposition, noise reduction

## Abstract

ECG signal processing is an important basis for the prevention and diagnosis of cardiovascular diseases; however, the signal is susceptible to noise interference mixed with equipment, environmental influences, and transmission processes. In this paper, an efficient denoising method based on the variational modal decomposition (VMD) algorithm combined with and optimized by the sparrow search algorithm (SSA) and singular value decomposition (SVD) algorithm, named VMD–SSA–SVD, is proposed for the first time and applied to the noise reduction of ECG signals. SSA is used to find the optimal combination of parameters of VMD [K,α], VMD–SSA decomposes the signal to obtain finite modal components, and the components containing baseline drift are eliminated by the mean value criterion. Then, the effective modalities are obtained in the remaining components using the mutual relation number method, and each effective modal is processed by SVD noise reduction and reconstructed separately to finally obtain a clean ECG signal. In order to verify the effectiveness, the methods proposed are compared and analyzed with wavelet packet decomposition, empirical mode decomposition (EMD), ensemble empirical mode decomposition (EEMD), and the complete ensemble empirical mode decomposition with adaptive noise (CEEMDAN) algorithm. The results show that the noise reduction effect of the VMD–SSA–SVD algorithm proposed is the most significant, and that it can suppress the noise and remove the baseline drift interference at the same time, and effectively retain the morphological characteristics of the ECG signals.

## 1. Introduction

In recent years, cardiovascular diseases have caused great harm to the human heart, and their lethality has posed a threat to human health, which is now being taken seriously. In clinical medicine, ECG signals record the electrical activity characteristics of the heart, and can detect abnormalities in the heart, and can be used as an important reference basis for the prevention and diagnosis of cardiovascular diseases. However, ECG signals are weak and of low frequency; the acquisition equipment, environmental changes, and the human body’s own activities are inevitably affected by noise; and the presence of noise causes certain difficulties in the identification of the characteristics of the ECG signals. The most serious impact is caused by baseline drift interference, which causes the baseline of ECG signals to deviate from the horizontal position, and can even cause the loss or distortion of signal characteristics. Therefore, how to separate the real ECG signals from the strong noise interference and retain the morphological features of the signals has become a key research direction in this field, and is of great importance.

As the level of signal processing becomes more mature, there are more new methods for ECG signal processing. The main noises in ECG signals are EMG interference, industrial frequency interference, and baseline drift. Because of the difficulty in removing baseline drift from ECG signals, it has the most serious impact on the characteristics of ECG signals, and it will be focused on in this paper.

To filter industrial frequency noise and myoelectric noise, in 2006, Zhao et al. combined empirical mode decomposition (EMD) with an infinite impulse response (IIR) trap to filter the industrial frequency noise in ECG signals [1]. Although there was a certain improvement in the denoising effect, the detailed information of the original signals could not be better preserved, and the mutation of the original signals could not be more accurately represented. In 2012, Kabir et al. combined the wavelet thresholding method and EMD to remove EMG noise [2]. The wavelet thresholding method improved the time–frequency resolution; however, the soft thresholding function compressed the function signal and made the function signal produce constant deviation, causing signal distortion as a consequence. In 2014, Choudhry et al. [3] used the wavelet thresholding method to filter EMG interference. However, the wavelet threshold denoising method needs to select the appropriate wavelet basis function, threshold function, threshold value, etc., and set the feasible number of decomposition layers according to the signal to be analyzed, which lacks a certain degree of adaptivity. In 2017, Xiao et al. decomposed ECG signals by EMD to obtain several IMF components, performed singular spectrum analysis (SSA) noise reduction on each IMF component, and finally reconstructed each IMF component to obtain the ECG signals with EMG interference removed [4]. Although this overcame the problem of wavelet basis function selection, the EMD algorithm suffers from the problems of modal confusion and endpoint effect, so the signal denoising effect was poor and the algorithm operation time was too long. In 2020, Yin et al. used ensemble empirical mode decomposition (EEMD) to complete the decomposition of noisy signals and finally obtained ECG signals with the interference removed [5]. This method can improve the modal mixing to a certain extent and is somewhat improved compared to the EMD algorithm; however, the workload and the time required for the whole algorithm are large and not complete. In the same year, Fu et al. [6] introduced peak error on the basis of complete ensemble empirical mode decomposition (CEEMD) to adaptively determine the number of ensemble averages and complete industrial frequency interference removal. This method not only reduces the amount of integration, but also makes the reconstruction error close to zero and causes less noise residue. However, if the parameters of CEEMD are not selected appropriately, the IMF from each decomposition is different, and the signal decomposition generates randomness, and even generates the wrong components leading to noncompliance in IMF. In 2021, Yang et al. proposed an improved wavelet thresholding function containing two dynamic parameters [7]. The method overcomes the shortcomings of soft and hard thresholding functions, and the denoising effect is more ideal, but the computation is large, the efficiency is low, and the algorithm’s structure is more complex. In 2022, Chen et al. proposed a wavelet packet analysis combined with singular value difference for an ECG signal denoising algorithm [8].

For filtering baseline drift, in 2014, Pang et al. used a mathematical morphology method to remove baseline drift in ECG signals followed by R-wave localization, which improved the R-wave recognition rate by 0.5% to 5% compared to wavelet transform [9]. In the same year, Ding et al. proposed a filtering algorithm based on the combination of EMD and morphological filtering to remove baseline drift, which is more effective compared to the single EMD method or the morphological filtering method [10]. In 2015, Sharmna proposed a method to remove the baseline drift in ECG signals by Hilbert vibration decomposition (HVD) [11]. In the same year, Gupta et al. used multivariate empirical modal decomposition (MEMD) to remove the baseline drift in ECG signals [12]. In 2016, Satijia et al. proposed the use of sparse decomposition to remove baseline drift interference in ECG signals [13]. In the same year, Prabhakararao et al. used variable modal decomposition (VMD), where the component corresponding to the baseline drift is estimated based on the center frequency, and then this component is subtracted from the noisy signal to achieve baseline drift removal [14]. This method works well, but the optimal number of layers needs to be calculated by a priori estimation. In 2017, Singh et al. used the empirical wavelet transform (EWT) to remove baseline drift [15]. Experiments show that the method has strong adaptive and real-time performance, but the algorithm is inefficient. In 2018, Jain et al. used EMD to decompose the band noise signal, then selected the components with baseline drift by spectral analysis, and used a Savitzky–Golay filter to weaken the baseline drift in the components. Finally, the processed IMF and the rest of the IMF were reconstructed to obtain the ECG signals without baseline drift [16]. This method is susceptible to the influence of the reference signal and has poor robustness. In the same year, Sun et al. proposed a wavelet packet decomposition method for removing baseline drift and a modified wavelet thresholding method for denoising the original ECG signals [17]. In 2020, Amit et al. proposed a Fourier decomposition method (FDM) to separate both baseline drift and PLI from ECG signals and obtain clean ECG data [18]. In 2021, Romero et al. proposed a filtering algorithm for ECG signals containing baseline drift using deep learning, which requires a large amount of experimental data [19].

In this paper, the latest intelligent optimization algorithm, the sparrow search algorithm (SSA), is applied to the parameter combination search of VMD, and is then combined with the SVD algorithm to form the final VMD–SSA–SVD noise reduction method. To verify its feasibility and superiority, the VMD–SSA–SVD denoising algorithm is applied to ECG signals and compared with other methods. The experimental results show that it can achieve the correction of baseline drift and the filtering of random noise interference, while retaining the complete characteristics of the signals.

## 2. Basic Knowledge of ECG Signals

### 2.1. Mechanism of ECG Signal Generation

**Figure 1:**
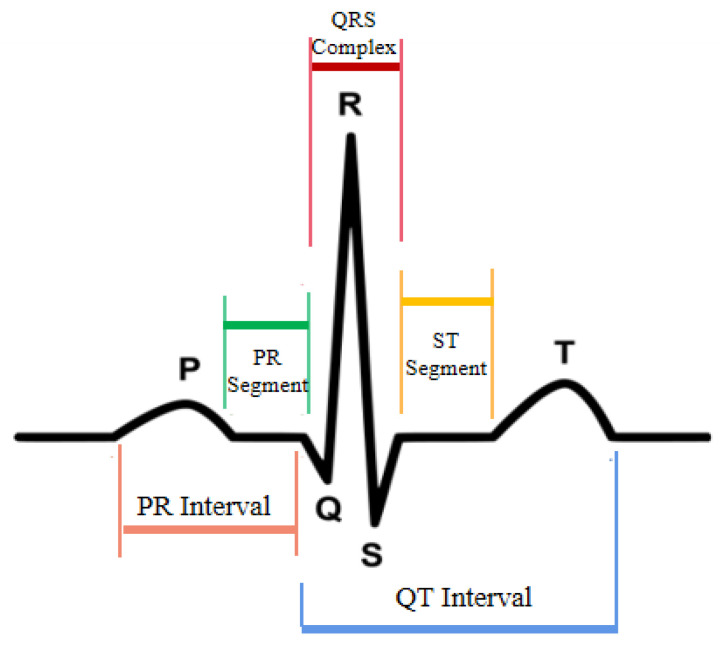
Waveform of standard heartbeat in one cycle.

The heart is the core organ of the human body, and with regular activity, it can continuously provide power for blood circulation. Along with the regular activity of the heart, ECG signals are microscopically expressed as the conduction of cardiomyocyte action potential, i.e., the action process of recording the electrical excitation of cardiomyocytes once. The ECG in clinical medicine is a visualization of the collected ECG signals, which are in the form of waves, segments, and intervals, each of which corresponds to the physiological activity of different parts of the heart and contains a specific medical meaning. Figure 1 shows the ECG waveform of a standard heartbeat during a cycle, which consists of a P-wave, QRS-wave, T-wave, QT-interval, PR-interval, PR-segment, and ST-segment.

### 2.2. Analysis of ECG Signal Noise

ECG signals are subject to noise interference from the environment, respiratory motion, the intensity of vital activity, and the amount of exercise in the subject’s body during the process of acquisition and transmission. The acquisition apparatus and the external environment, which is always changing, also cause some physical phenomena, such as electromagnetic emission, which inevitably introduce a certain degree of noise interference.

In this paper, we used the MIT-BIH ECG database, which was jointly established by the Massachusetts Institute of Technology and Beth Israel Hospital [20]. Each ECG signal in it contains information related to heartbeat, R-wave position, heartbeat type, and signal quality, individually labeled by several medical experts. The database also specifically records diagnostic information about normal or abnormal signals labeled by cardiologists.

**Figure 2:**
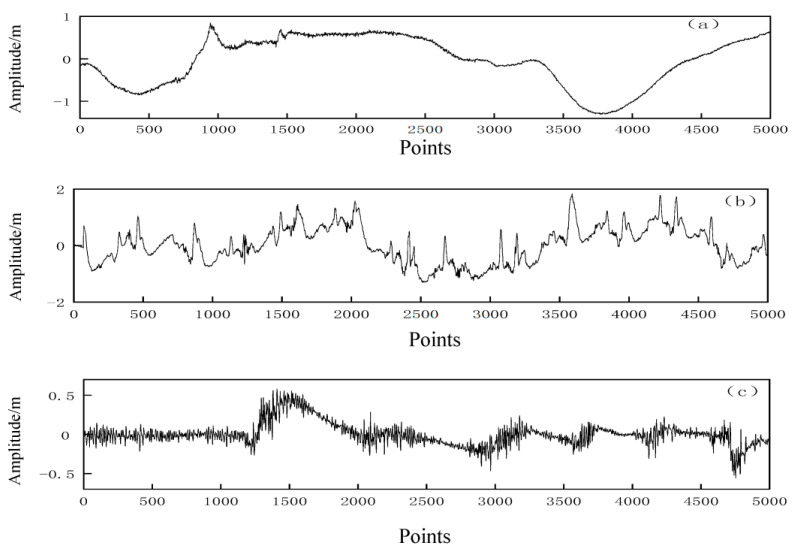
Waveform plots of three types of noise. (**a**) Baseline drift, (**b**) electrode activity interference, and (**c**) myoelectric interference.

The analysis of noise interference revealed three main types of noise in general: baseline wander (BW), muscle artifact (MA), and electrode motion (EM) artifacts [21]. The waveform plots for each of the three types of noise sources, including baseline drift, electrode activity interference, and myoelectric interference, are shown in Figure 2.

Among them, the baseline drift has the most serious impact on ECG signals, and due to its low frequency, the baseline drift partially overlaps with the ST-segment of the ECG signal in the frequency band [22], which causes distortion in the signal waveform after denoising. Additionally, the correction of baseline drift is a prerequisite for the accurate extraction of the characteristic information of ECG signals.

## 3. Methodology

### 3.1. VMD Algorithm

The VMD algorithm is adaptive and non-recursive for signal processing [23]. The theoretical framework of the algorithm is to search for the optimal solution of the entire variational model through several iterations, then determine the center frequency and bandwidth of each intrinsic mode component, and finally decompose the IMF components with a predetermined number of scales of *k* fixed bandwidths to achieve an effective dissection in the frequency domain of the signal. The VMD method has many advantages, such as high decomposition accuracy, fast computational speed, strong theoretical support, and high robustness, due to its own Wiener filtering characteristics.

The parameters of the VMD algorithm are closely related to its decomposition performance, including the modal number K, quadratic penalty factor α, fidelity coefficient τ, and convergence tolerance ε. In the process of decomposing different signals, these parameters need to change adaptively to achieve the optimal decomposition effect. On the premise of avoiding modal mixing, the effective parameter setting has a great influence on the decomposition effect of the whole signal. In fact, the combined study found that K and α in the VMD algorithm have a particularly significant effect on the decomposition effect, while τ and ε have little effect on the decomposition result, and the standard default value is generally used.

### 3.2. SSA Algorithm

SSA, a new swarm intelligence optimization algorithm, has a bionic principle that simulates the foraging and anti-predation behavior of sparrows [24]. In this paper, in view of the SSA advantages such as fewer parameter adjustments, strong robustness, and easy implementation, as well as the VMD disadvantage of difficult parameter selection, the SSA algorithm is applied to the parameter optimization of VMD to achieve the adaptive selection of VMD parameters, which to some extent makes up for its deficiencies, thus obtaining the optimal parameter combination [K,α].

Considering that the SSA algorithm needs to determine an adaptation function when searching for parameters, the adaptation value of individual sparrows is calculated each time their positions are updated, comparing the adaptation values of each position in the population [25]. In order to search for the best global component, the local minimal entropy value is used as the fitness function in the optimization search process.

Compared with the traditional optimization algorithm, the SSA algorithm has a great advantage both in finding the best component and in iteration speed. The VMD–SSA method requires determining the range of the VMD parameters K and α, and setting the model parameters of SSA. In this paper, K takes an integer in the interval [2, 15]; α takes an integer in the interval [500, 5000]; and the maximum number of iterations is set to 15. The number of populations is set to 30. As the number of current iterations increases, the fitness values at each position are continuously calculated and compared for updating until after they are greater than the maximum number of iterations. Finally, the best fitness value of the population and the optimal parameters K and α are output, and we save the values.

In this paper, in order to test the performance of the SSA algorithm to optimize the VMD decomposition, and to demonstrate the strong local optimization-seeking ability of SSA, a set of test signals were selected for verification. The composition of the signals is shown in Equation (1).

(1) $$\begin{array}{l} f\left(t\right)=x_{1}\left(t\right)+x_{2}\left(t\right)+x_{3}\left(t\right)+\delta \\ \;\;=sin\left(2\pi *6*t\right)+1.2\cos\left(2\pi *55*t\right)+1.4sin\left(2\pi *180*t\right)+\delta \end{array}$$

In order to verify the superiority of the decomposition ability of the VMD–SSA algorithm, different intelligent optimization algorithms were selected to optimize the parameter combinations of VMD and compare their decomposition effects with the VMD–SSA algorithm. Among many traditional optimization algorithms, three optimization algorithms, the representative particle swarm optimization algorithm (PSO), gray wolf optimization algorithm (GWO), and whale optimization algorithm (WOA), were selected. The VMD decomposition of the same test signal was performed by the four optimization algorithms based on the same parameters set, and the differences in the optimization aspects of the four algorithms were finally compared. The optimal combination of parameters for each of the four optimization algorithms is listed in Table 1.

**Figure 3:**
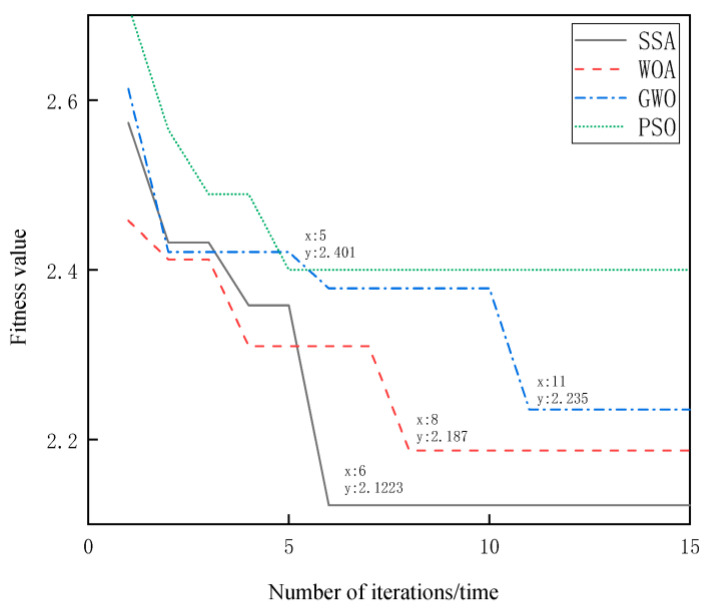
Fitness value curves of different optimization algorithms.

The curve of the fitness value of the four optimization algorithms with the number of iterations is shown in Figure 3. From the figure, it can be observed that the convergence values of the fitness functions of the different optimization algorithms vary, among which the best fitness value of the SSA algorithm is relatively the smallest among the four algorithms, converging at 2.1223.

Comparing the convergence speed of the four optimization algorithms from the convergence curves of the fitness values, it can be seen that PSO completes the global search the fastest, and the curve smooths out at the fifth iteration. However, the decomposition result of the VMD–PSO algorithm is the least satisfactory; it does not separate the noise component effectively, and its decomposition ability performs poorly compared with the other optimization algorithms. This is followed by SSA converging in the sixth generation, WOA converging in the eighth generation, and GWO converging in the eleventh generation. The decomposition results of the VMD–SSA algorithm have a single-center frequency for each component of the test signal and do not interfere with each other, and the useful signal components are well separated from the noise components, so the global search effect of the SSA algorithm optimizing the VMD parameter combination is the best.

### 3.3. Selection of Effective Components Based on the Number of Interrelationships

To extract the signal feature information more accurately, the interrelation number method was used to select the effective IMF components, and the amount of correlation information between two components was measured, which is more accurate compared with the traditional correlation coefficient selection method. To achieve the selection of effective IMF components, we found the number of interrelationships between each IMF component and the original signal after decomposition, calculated the threshold value by the formula, and compared each correlation coefficient with its threshold value to select the component greater than the threshold value as the effective component. The number of interrelationships and the threshold value are calculated as

(2) $$\rho_{xy}=\frac{\sum_{n=1}^{N}\left[\left(x_{n}-\bar{x}\right)\left(y_{n}-\bar{y}\right)\right]}{{\left[\sum_{n=1}^{N}{\left(x_{n}-\bar{x}\right)}^{2}\sum_{n=1}^{N}{\left(y_{n}-\bar{y}\right)}^{2}\right]}^{\frac{1}{2}}}$$

(3) $$\mu =\frac{\max\left(\rho_{xy}\right)}{10\times \max\left(\rho_{xy}\right)-3}$$

where $\max\left(\rho_{xy}\right)$ is the maximum correlation coefficient, $x_{n}$ is the IMF component, $y_{n}$ is the original signal, and $\bar{x}$ and $\bar{y}$ are the mean values of the IMF component and the original signal, respectively.

### 3.4. SVD Algorithm

Sanliturk et al. introduced the Hankel matrix for SVD, and the results showed its ability to extract the effective signal from the noise to an acceptable degree [26]. In its subsequent development and improvement, it gradually became widely used. The principle of SVD noise reduction is as follows: first, the original noisy signal is constructed into a Hankel matrix *H* of order p×q; then, the SVD is used to decompose the matrix *H* into singular values to obtain the singular value matrix of the signal $\sigma_{i}$. Finally, the singular values corresponding to the noise are set to zero, and then the signal is reconstructed using the SVD inverse operation to obtain the noise-reduced signal.

The SVD decomposition of the Hankel matrix *H* yields

(4) $$H=U\sum V^{T}=U_{s}\sum_{s}V_{s}^{T}=U_{n}\sum_{n}V_{n}^{T}=\sum_{i=1}^{l}\sigma_{i}u_{i}v_{i}^{T}$$

where $\sigma_{i}$ is the singular value corresponding to the demarcation between the real signal and the noisy signal [27]. In the SVD noise reduction process, the most critical step is to select the component matrices that match the characteristics of the original signal for reconstruction. Selecting fewer matrix components in the reconstruction process leads to serious distortion in the reconstructed signal, while selecting more matrix components results in too much noise in the reconstructed signal. In this paper, we use the singular value difference spectrum method to select the appropriate matrix components to reconstruct the noise-reduced signal.

### 3.5. VMD–SSA–SVD Noise Reduction Method

ECG signals are characterized by being nonstationary and low frequency, and having weak anti-interference ability. Additionally, the ECG signal itself is susceptible to noise interference generated by the human body or external environmental factors, among which the effect of baseline drift is especially obvious, as it causes difficulties in subsequent signal detection. Therefore, removing baseline drift and suppressing noise interference are the most critical steps in ECG signal preprocessing.

In this paper, the proposed noise reduction algorithm based on VMD–SSA–SVD was applied to real ECG signals from the MIT-BIH database. Firstly, the SSA algorithm was used to find the globally optimal combination of parameters [K,α] in the VMD; then, a series of IMF components were obtained by effectively decomposing the signal according to the VMD–SSA method, and the mean value of each IMF component was counted. Any IMF component with a mean value greater than a set threshold means that the component is associated with the baseline drift signal. The estimated baseline drift was extracted and directly removed from the original signal; then, the interrelationship between the original signal and each IMF component and the threshold value were calculated; the effective IMF component and the high-frequency noise component were discriminated based on the threshold value setting; the effective IMF component was retained; and the high-frequency noise component was rejected. Because the effective IMF components still contained a small amount of noise, the effective IMF components were further converted into Hankel matrices, and then the SVD noise reduction process was performed to determine the effective order based on the position of the maximum peak in the singular value difference spectrum to reconstruct the noise-reduced IMF components. The effective IMF components were reconstructed after the noise reduction process to finally obtain the noise-reduced ECG signal, achieving the baseline correction and random noise removal in the noisy ECG signal. In addition, the noise reduction performance of the algorithm proposed in this paper was compared with that of the wavelet packet decomposition algorithm, EMD algorithm, EEMD algorithm, and complete ensemble empirical mode decomposition with adaptive noise (CEEMDAN) algorithm, and the effectiveness and superiority of the algorithm in this paper were verified by the noise reduction effect graphs and evaluation indices of the five algorithms.

## 4. Results and Discussion

### 4.1. Evaluation Index of the Noise Reduction Effect

For evaluating and verifying the effectiveness of noise reduction, two reference indicators were used: signal-to-noise ratio (SNR) and mean square error (MSE). SNR refers to the power ratio between the signal and noise, reflecting the quality of the signal. The higher the value, the better the denoising effect. MSE refers to the variance between the original signal and the denoised signal, reflecting the similarity between the two signals before and after denoising, and the smaller the value, the higher the similarity. The SNR and MSE are defined by

(5) $$SNR=10\log\left(\frac{\sum_{i=1}^{N}x_{i}{}^{2}}{\sum_{i=1}^{N}{\left(x_{i}-\tilde{x_{i}}\right)}^{2}}\right)$$

(6) $$MSE=\frac{1}{N}\sum_{i=1}^{N}{\left(x_{i}-\tilde{x_{i}}\right)}^{2}$$

where $x_{i}$ is the original signal, ${\tilde{x}}_{i}$ is the denoised signal, and *N* is the signal length.

### 4.2. Simulated Noise-Containing ECG Signal Validation

In order to verify the feasibility of the proposed noise reduction algorithm, the pure 103 ECG signal superimposed with analog baseline drift, and analog noise from the MIT-BIH database was selected as the test signal to be processed, where the analog baseline drift is a sinusoidal signal with an amplitude of 0.4 mV and a frequency of 0.5 Hz, the analog noise is a Gaussian white noise with a specified SNR of 10 dB, and the test signal is noise reduced by the EMD, EEMD, CEEMDAN, wavelet packet decomposition, WOA–VMD, and VMD–SSA–SVD algorithms, respectively. In this paper, the analyses were performed from two perspectives: Firstly, from a qualitative perspective, the noise reduction effects were compared visually to qualitatively evaluate the differences in the noise reduction performance of the five algorithms. Secondly, from a quantitative perspective, the quantitative indices, such as SNR and MSE, were used to objectively and quantitatively analyze the differences in the noise reduction performance of each algorithm.

**Figure 4:**
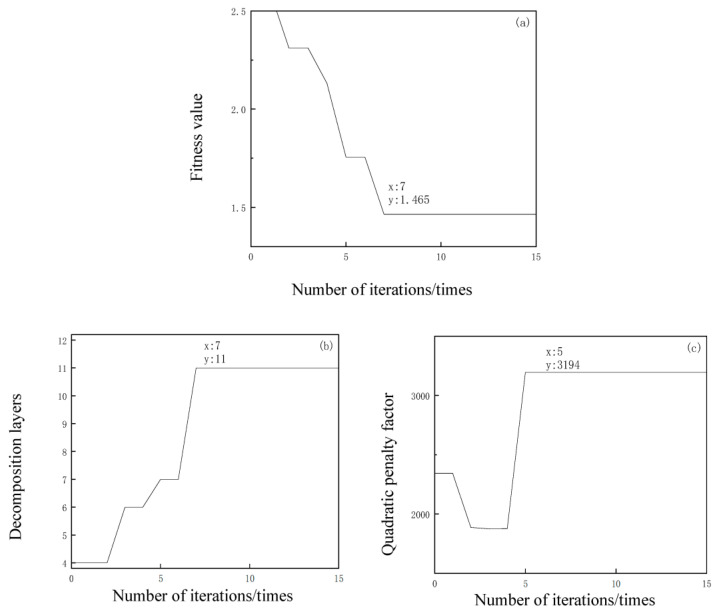
Parameter optimization and fitness iteration process of VMD–SSA–SVD. (**a**) Fitness value. (**b**) Convergence of decomposition layers. (**c**) Convergence of the quadratic penalty factor.

The wavelet packet noise reduction method uses a db9 wavelet basis function for 3-layer decomposition, and the EEMD method adds white noise with a standard deviation of 0.2 and averages the values 100 times. Moreover, the fitness values, parameters K and α of the VMD–SSA–SVD algorithm, and the number of iterations are shown in Figure 4. It is clear that the optimal value of the number of decomposition layers K is 11, as shown in Figure 4b, and the optimal value of the penalty factor α is 3194, as shown in Figure 4c, so the optimal combination of the corresponding VMD decomposition parameters is [11, 3194].

**Figure 5:**
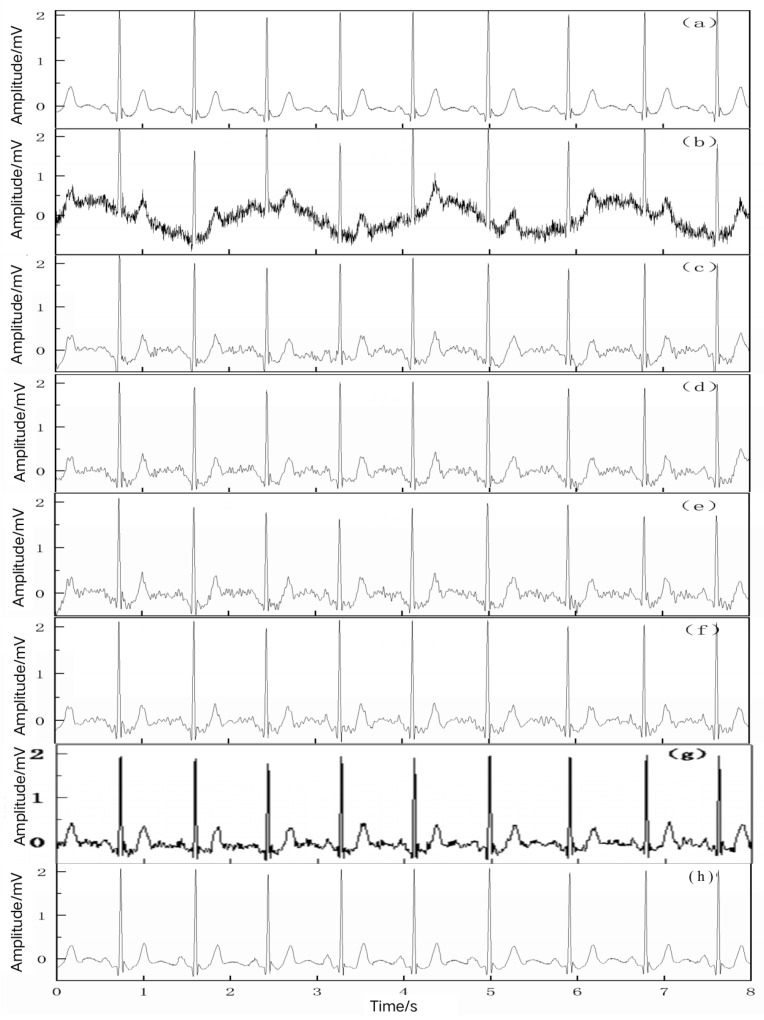
Noise reduction effect of 103 simulated signals containing noise. (**a**) The original 103 signal, (**b**) the simulated 103 signal containing noise, (**c**) effect of noise reduction by EMD, (**d**) effect of noise reduction by EEMD, (**e**) effect of noise reduction by CEEMDAN, (**f**) effect of noise reduction by wavelet threshold, (**g**) effect of noise reduction by WOA–VMD, and (**h**) effect of noise reduction by VMD–SSA–SVD.

Figure 5 shows the noise reduction effect of the 103 simulated mixed signals. As seen in Figure 5, these six denoising methods have a certain degree of improvement for the removal of baseline drift and the suppression of analog noise interference.

Figure 5b shows the 103 simulated signal containing noise, which shows that the presence of baseline drift causes the baseline of the ECG signal to not be in a horizontal position, and the presence of noise interference makes the waveform characteristics of the ECG signal completely masked.

Figure 5c shows the signal after being denoised by the EMD method. It can be seen that there is a slight baseline drift in the signal, and the PR segment does not remain in its horizontal position and still has fluctuations, which also causes the amplitude of the R- and T-waves in the signal to be different. Due to the defect of modal mixing in EMD, the T- and P-waves between two adjacent heartbeats are still elevated and contain a small amount of noise interference, and there is also unfiltered noise in the vicinity of the QRS-wave group.

Figure 5d shows the signal after being denoised by the EEMD method. As seen in Figure 5d, the baseline drift is better corrected than by the EMD method, but the R-wave amplitude is attenuated compared with the original signal, and the amplitude of the signal at the eighth is higher than that of the original ECG signal. Due to this, inevitably, the T-P segment between the two beats is still elevated and contains noise interference, and the noise in the vicinity of the QRS group is still not filtered out.

Figure 5e shows the signal after being denoised by the CEEMDAN method, showing that the waveform is shifted down at the starting point, and the R-wave amplitude of the signal is severely reduced, while the T-wave amplitude is higher than that of the original signal. The most obvious effect is that the waveform shape of the P-wave is not well recognized.

Figure 5f shows the signal after being denoised by the wavelet threshold method. Compared with the above methods, the noise in the T-P segment and QRS-wave group is slightly improved, the amplitude of the R- and T-waves is closer to that of the original signal, and the morphological features of the T- and P-waves can be recognized. However, the signal waveform is still not smooth enough, and the waveform uplift phenomenon of the T-P segment is still not effectively improved, especially near the first few T-waves; the later segments shift upward.

Figure 5g shows the signal after being denoised by the WOA–VMD method. It can be seen that the signal waveform is shifted upward at the starting point, and the T-P band between two adjacent heartbeats is still mixed with noise interference, meaning the waveform shape of T- and P-waves cannot be seen clearly, and there is still a small amount of noise near the QRS-wave group.

Figure 5h shows the signal after being denoised by the VMD–SSA–SVD method. As seen in Figure 5g, the baseline drift of the signal is well corrected, the overall baseline is at a horizontal position, the signal waveform is smoother, and the noise interference is well removed compared with the other methods. The T-P segment maintains the morphological characteristics of the original signal, and there is no obvious upward phenomenon. The amplitude of each wave of the processed signal is nearly the same as that of the original amplitude, the morphological recognition of each wave is good, and the distortion among each wave is reduced. The processed signal is basically consistent with the original ECG signal in terms of reproduction.

In summary, the final noise reduction effects of different denoising methods, shown in Figure 5, mean that the VMD–SSA–SVD method exhibits superior noise reduction performance in terms of the correction of baseline drift and noise suppression.

The noise reduction performance of different algorithms was analyzed from a qualitative perspective, but these subjective judgments may bring errors. In order to objectively analyze the noise reduction effect, some quantitative analyses for different algorithms were performed through SNRs and MSEs, as shown in Table 2. As seen in Table 2, the VMD–SSA–SVD method proposed has the highest SNR and the lowest MSE compared with the other four methods, which proves that the VMD–SSA–SVD method has the best noise reduction effect, and the signal after noise reduction is closer to the real ECG signal.

**Table 2:** The SNRs and MSEs of signals denoised by the five methods shown in Figure 5.

Denoising Algorithm	SNR (dB)	MSE
**EMD**	12.8600	0.0642
**EEMD**	14.6328	0.0743
**CEEMDAN**	13.4756	0.0792
**Wavelet threshold**	16.7885	0.0431
**WOA–VMD**	16.7885	0.067
**VMD–SSA–SVD**	19.74	0.0269

### 4.3. Testing Real Signals with Base Drift

**Figure 6:**
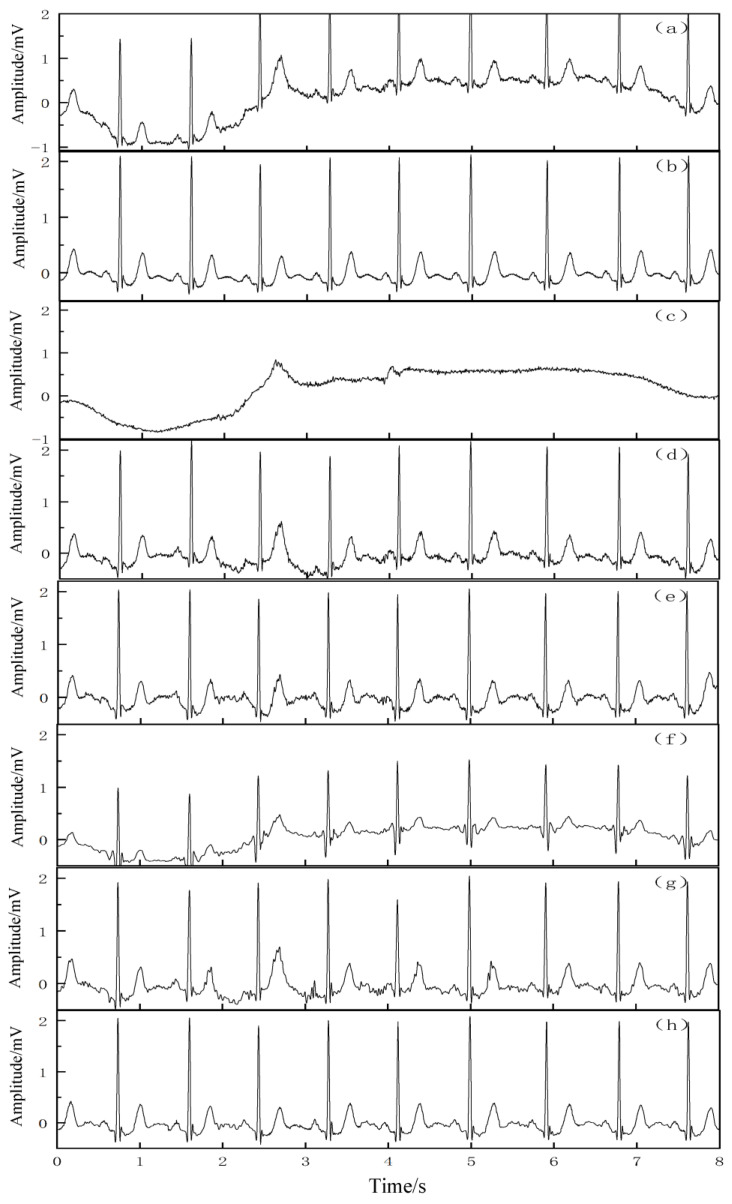
Noise reduction effect of 103 signal with real base drift. (**a**) The 103 signal with real base drift, (**b**) original 103 signal, (**c**) real base drift, (**d**) effect of noise reduction by EMD, (**e**) effect of noise reduction by EEMD, (**f**) effect of noise reduction by wavelet threshold, (**g**) effect of noise reduction by CEEMDAN, and (**h**) effect of noise reduction by VMD–SSA–SVD.

**Figure 7:**
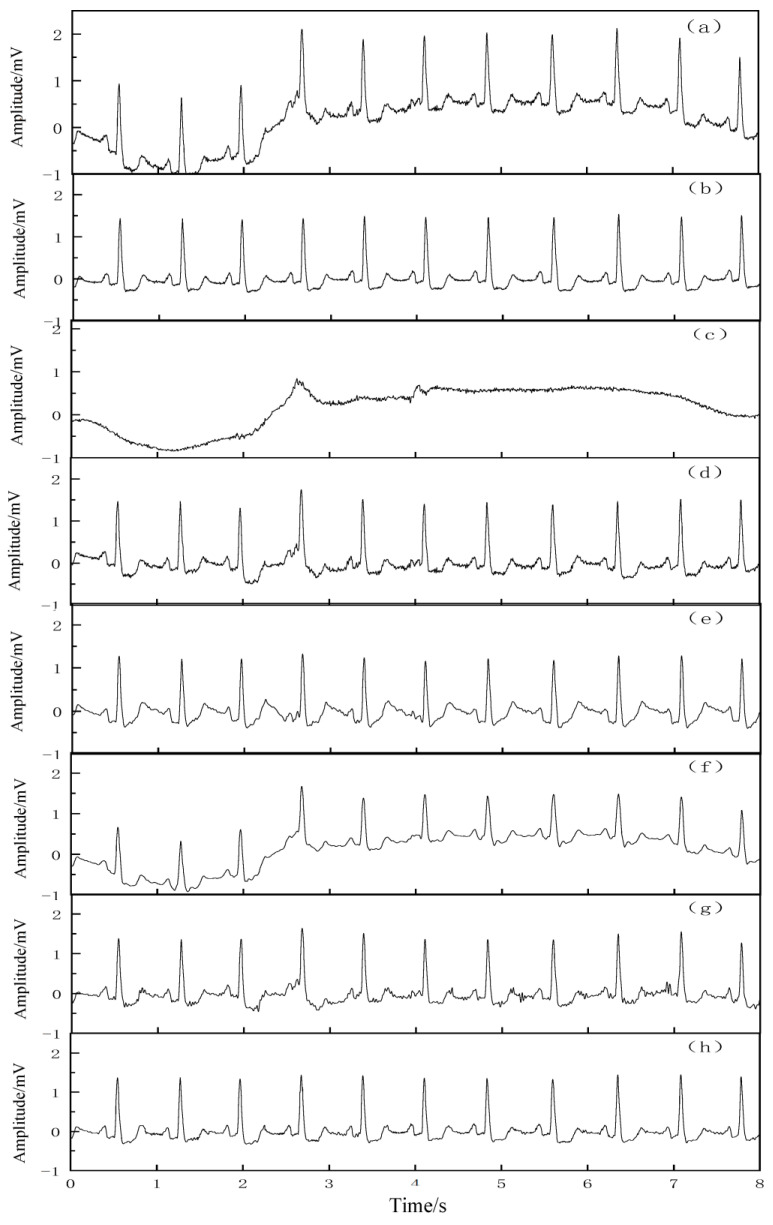
Noise reduction effect of 105 signal with real base drift. (**a**) The 105 signal with real base drift, (**b**) original 105 signal, (**c**) real base drift, (**d**) effect of noise reduction by EMD, (**e**) effect of noise reduction by EEMD, (**f**) effect of noise reduction by wavelet threshold, (**g**) effect of noise reduction by CEEMDAN, and (**h**) effect of noise reduction by VMD–SSA–SVD.

In order to verify the removal effect of the proposed algorithm on the real baseline drift, 2 sets of more pure ECG signals were selected from the MIT-BIH library, namely 103 and 105 signals, and the real baseline drift (bw-noise1) from the MIT-BIH noise stress database was superimposed on these 2 sets of ECG signals to form a new simulation signal. The two sets of signal tests were performed by the EMD, EEMD, wavelet packet decomposition, CEEMDAN, and VMD–SSA–SVD algorithms, respectively. Figure 6 and Figure 7 show the removal reduction effect of the 103 and 105 signals with real baseline drift, respectively. The qualitative analysis showed that all five denoising methods improved the real baseline drift and noise interference to some extent. The morphological features and details of the signal waveforms were almost completely preserved.

In the quantitative analysis, the SNRs and MSEs of the signals after noise reduction were calculated for each algorithm, as listed in Table 3. As seen in Table 3, the proposed VMD–SSA–SVD method has the highest SNR and the lowest MSE compared with the other four methods, which proves that its removal effect on the real baseline drift is better than that of the other four methods.

### 4.4. Actual Data Testing

**Figure 8:**
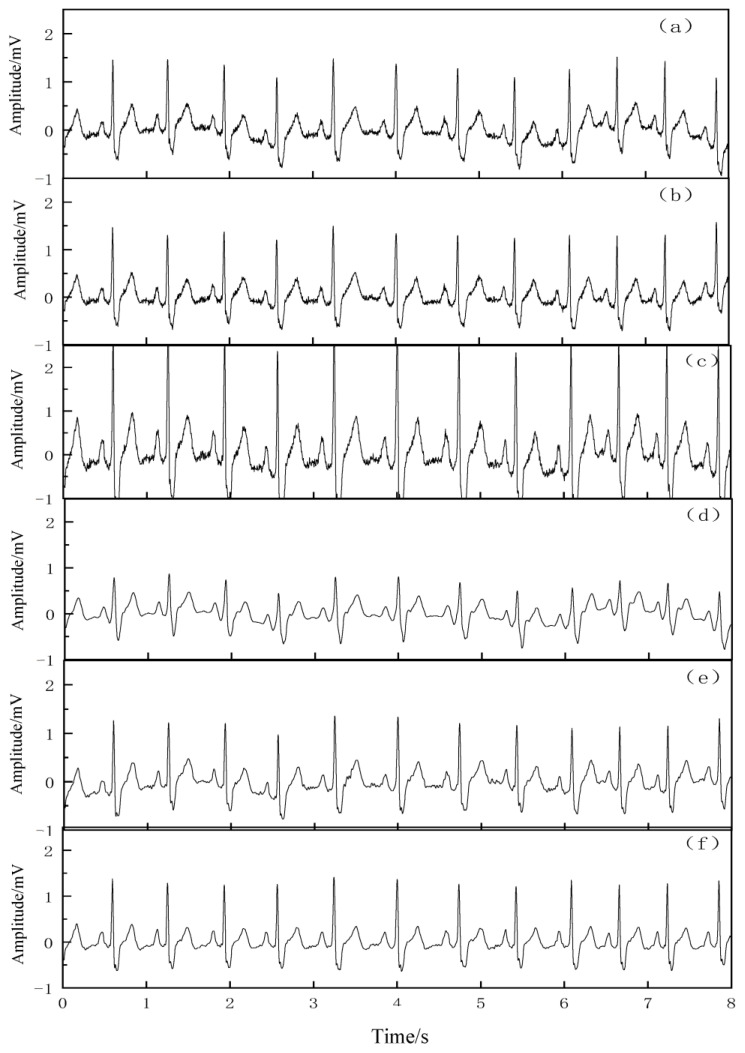
Noise reduction effect of actual 212 ECG signal. (**a**) Original 212 signal, (**b**) effect of noise reduction by EMD, (**c**) effect of noise reduction by EEMD, (**d**) effect of noise reduction by wavelet threshold, (**e**) effect of noise reduction by CEEMDAN, and (**f**) effect of noise reduction by VMD–SSA–SVD.

**Figure 9:**
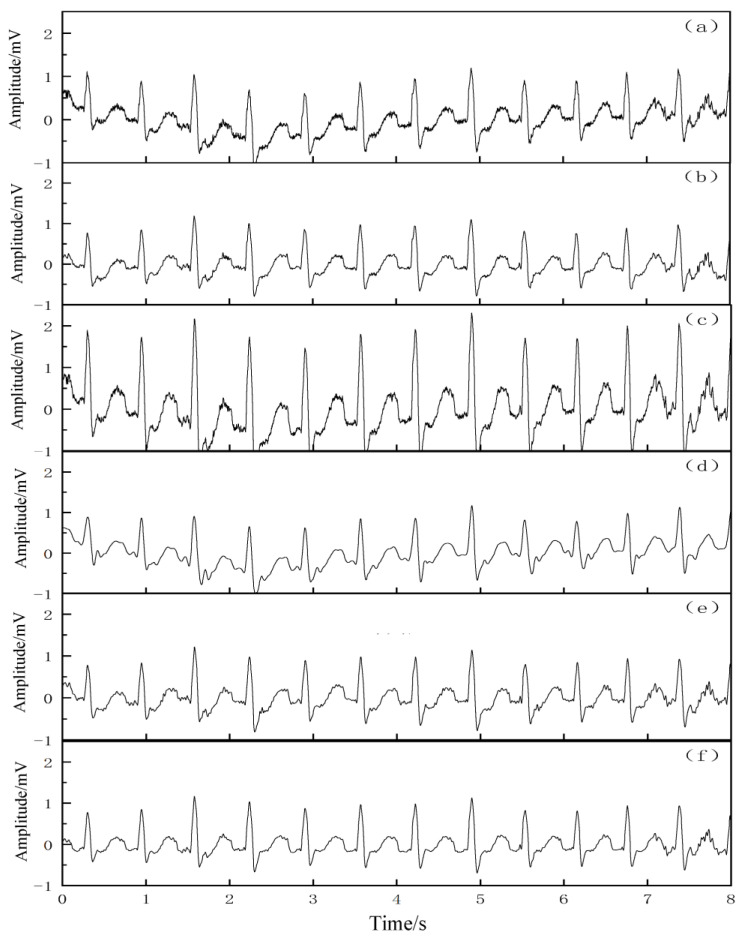
Noise reduction effect of actual 109 ECG signal. (**a**) Original 109 signal, (**b**) effect of noise reduction by EMD, (**c**) effect of noise reduction by EEMD, (**d**) effect of noise reduction by wavelet threshold, (**e**) effect of noise reduction by CEEMDAN, and (**f**) effect of noise reduction by VMD–SSA–SVD.

In order to further prove the effectiveness of the algorithm applied to the actual ECG signals, 2 sets of representative actual ECG signals were selected from the MIT-BIH database, namely 212 and 109 signals. Figure 8 and Figure 9 show the noise reduction effects of the 212 and 109 ECG signals, respectively.

As seen in Figure 8 and Figure 9, these two groups of ECG signals have different waveform morphologies, and both groups of signals have different degrees of baseline drift and random noise interference. From the qualitative point of view, the proposed VMD–SSA–SVD algorithm can preserve the details of the original signal, and the morphological characteristics of the noise-reduced signal are well reproduced from the original signal, meaning that the processed signal not only corrects the baseline drift but also filters out the noise interference, indicating that the noise reduction performance of the VMD–SSA–SVD algorithm is superior. Since the SNR of the original ECG signal is not known, and the SNR of the baseline drift and noise contained in the signal is not known, the SNR and MSE of the signal after noise reduction by each algorithm cannot be exactly derived from a quantitative analysis.

## 5. Conclusions

In this paper, the VMD–SSA–SVD-based denoising method was proposed and applied to the noise reduction of ECG signals to achieve the correction of baseline drift and the suppression of random noise interference. Firstly, VMD–SSA decomposition was performed on a noisy ECG signal to find the optimal combination of parameters, and the components containing baseline drift were selected from all IMF components after the decomposition and subtracted from the original signal. Then, the effective IMF components were selected from the remaining components. A Hankel matrix was constructed for each effective IMF component, and the SVD noise reduction process was performed separately. Three types of noisy ECG signals, namely simulated signals with simulated basis drift and simulated noise, simulated signals with real basis drift, and actual ECG signals, were used for validation, and the advantages and disadvantages of each noise reduction method were analyzed. The VMD–SSA–SVD has the highest SNR and the smallest MSE, and its denoising effect and signal fidelity are better than those of the other four methods, which shows its effectiveness, superiority, and strong adaptability in ECG signal denoising. Therefore, the proposed VMD–SSA–SVD-based denoising method can achieve the correction of baseline drift and the filtering of random noise interference simultaneously, while retaining the complete characteristics of the signal, which is a novel method for ECG signal noise reduction. Regarding future improvements, the optimization algorithm takes a slightly longer time for the global search of optimal parameter combinations, so the computing efficiency needs to be further improved. Additionally, the measured ECG signals of various patients in hospitals should be collected in large numbers so that the ECG signals of different patients with different characteristics can be selected as test signals to further verify the universality of the denoising method in the application of ECG signal denoising.

## Figures and Tables

**Table 1:** Comparison of VMD optimization based on different optimization algorithms.

Optimization Algorithm	GWO		PSO		WOA		SSA	
**VMD Parameters**	K	α	K	α	K	α	K	α
**Parameter Value**	5	2046	4	1675	5	2458	4	2779

**Table 3:** Performance comparison of five denoising algorithms.

Signal	EMD		EEMD		CEEMDAN		Wavelet Threshold		VMD–SSA–SVD	
	SNR (dB)	MSE	SNR (dB)	MSE	SNR (dB)	MSE	SNR (dB)	MSE	SNR (dB)	MSE
**103**	11.632	0.103	15.654	0.074	13.25	0.096	10.213	0.168	18.476	0.043
**105**	13.375	0.982	17.221	0.087	15.452	0.098	11.468	0.160	19.69	0.038

## Data Availability

The relevant data used to support the findings of this study are available from the corresponding author upon request.

## References

[1] Zhao Z.D., Chen Y.Q. A new method for removal of baseline wander and power line interference in ECG signals. Proceedings of the International Conference on Machine Learning and Cybernetics.

[2] Kabir M.A., Shahnaz C. (2012). Denoising of ECG signals based on noise reduction algorithms in EMD and wavelet domains. Biomed. Signal Process. Control.

[3] Chaudhary M.S., Kapoor R.K., Sharma A.K. Comparison between different wavelet transforms and thresholding techniques for ECG denoising. Proceedings of the 2014 International Conference on Advances in Engineering & Technology Research (ICAETR-2014).

[4] Xiao X.B., Liu H.L., Ma Z.J. (2017). An empirical modal decomposition denoising method based on singular spectrum analysis. Comput. Eng. Sci..

[5] Yin L., Chen F.M., Zhang Q., Chen X. (2020). A cardiac adaptive denoising method using ensemble empirical modal decomposition and improved threshold function. J. Xi’an Jiaotong Univ..

[6] Fu L.J., Wang F.S., Liu Z.N. (2020). Application of improved adaptive CEEMD method for ECG signal denoising. J. Electron. Meas. Instrum..

[7] Yang C.J., Nie C.Y., Wang H.Y., Ruan X.L. (2021). Research and effect evaluation of myoelectric interference noise reduction based on wavelet improvement threshold. Electron. Meas. Technol..

[8] Chen S.Y., Zhang B.W., Liu X.M. (2022). Wavelet packet analysis combined with singular value differencing for ECG signal denoising algorithm. J. Nanjing Norm. Univ. (Nat. Sci. Ed.).

[9] Pang Y., Deng L., Lin J.Z. (2014). A morphological filtering-based method for removing baseline drift from ECG signals. J. Phys..

[10] Ding R., Li G.J., Wang Q. (2014). Research on baseline drift removal method for ECG signals. J. Yunnan Univ. (Nat. Sci. Ed.).

[11] Sharma H., Sharma K.K. (2015). Baseline wander removal of ECG signals using Hilbert vibration decomposition. Electron. Lett..

[12] Gupta P., Sharma K.K., Joshi S.D. (2015). Baseline wander removal of electrocardiogram signals using multivariate empirical mode decomposition. Healthc. Technol. Lett..

[13] Satija U., Ramkumar B., Manikandan M.S. A robust sparse signal decomposition framework for baseline wander removal from ECG signal. Proceedings of the 2016 IEEE Region 10 Conference (TENCON).

[14] Prabhakararao E., Manikandan M.S. On the use of variational mode decomposition for removal of baseline wander in ECG signals. Proceedings of the 2016 Twenty Second National Conference on Communication (NCC).

[15] Singh O., Sunkaria R.K. (2017). ECG signal denoising via empirical wavelet transform. Australas. Phys. Eng. Sci. Med..

[16] Jain S., Bajaj V., Kumar A. (2018). Riemann liouvelle fractional integral based empirical mode decomposition for ECG denoising. Biomed. Health Inform..

[17] Sun Y.N., Lv K.J., Zhang R. (2018). A new R-peak method for ECG signal. A new method for automatic detection of R-peaks in ECG signals. J. Northwestern Univ. (Nat. Sci. Ed.).

[18] Amit S., Pushpendra S., Binish F., Pachori R.B. (2020). An efficient removal of power-line interference and baseline wander from ECG signals by employing Fourier decomposition technique. Biomed. Signal Process. Control.

[19] Romero F.P., Piñol D.C., Vázquez-Seisdedos C.R. (2021). DeepFilter: An ECG baseline wander removal filter using deep learning techniques. Biomed. Signal Process. Control.

[20] Moody G.B., Mark R.G. (2001). The impact of the MIT-BIH arrhythmia database. IEEE Eng. Med. Biol. Mag. Q. Mag. Eng. Med. Biol. Soc..

[21] Satija U., Ramkumar B., Manikandan M.S. (2018). Automated ECG Noise Detection and Classification System for Unsupervised Healthcare Monitoring. IEEE J. Biomed. Health Inform..

[22] Huang W.W., Cai N., Xie W., Ye Q., Yang Z. (2015). ECG Baseline Wander Correction Based on Ensemble Empirical Mode Decomposition with Complementary Adaptive Noise. J. Med. Imaging Health Inform..

[23] Zhao W. (2018). Early fault diagnosis of gearboxes based on VMD and FSK. Mech. Drives.

[24] Liu X.D., Zhang L., Zhang Z.R., Zhao T., Zou L. (2021). Ultra-short-term Wind Power Prediction Model Based on VMD Decomposition and LSTM. IOP Conf. Ser. Earth Environ. Sci..

[25] Tang G.J., Wang X.L. (2015). Application of parameter-optimized variational modal decomposition method in early fault diagnosis of rolling bearings. J. Xi’an Jiaotong Univ..

[26] Sanliturk K.Y., Cakar O. (2005). Noise elimination from measured frequency Response functions. Mech. Syst. Signal Process..

[27] Li N., Deng S., Tang Q.B. (2013). Speech Enhancement Algorithm Based on Combining Local Characteristic-Scale Decomposition and Difference Spectrum of Singular Values. J. Multimed..

